# YBX1 Enhances Metastasis and Stemness by Transcriptionally Regulating MUC1 in Lung Adenocarcinoma

**DOI:** 10.3389/fonc.2021.702491

**Published:** 2021-12-15

**Authors:** Qiang Xie, Shilei Zhao, Wenzhi Liu, Yanwei Cui, Fengzhou Li, Zhuoshi Li, Tao Guo, Wendan Yu, Wei Guo, Wuguo Deng, Chundong Gu

**Affiliations:** ^1^ Department of Thoracic Surgery, The First Affiliated Hospital of Dalian Medical University, Dalian, China; ^2^ Lung Cancer Diagnosis, and Treatment Center of Dalian, The First Affiliated Hospital of Dalian Medical University, Dalian, China; ^3^ Zhongshan Hospital, Dalian University, Dalian, China; ^4^ Institute of Cancer Stem Cell, Lung Cancer Diagnosis and Treatment Center, Dalian Medical University, Dalian, China; ^5^ State Key Laboratory of Oncology in South China, Collaborative Innovation Center of Cancer Medicine, Sun Yat-sen University Cancer Center, Guangzhou, China

**Keywords:** YBX1, MUC1, prognosis, transcriptional regulation, metastasis

## Abstract

Abnormal expression of the transcription factor Y-box-binding protein-1 (YBX1) is associated with the proliferation, migration, aggressiveness, and stem-like properties of various cancers. These characteristics contribute to the tumorigenesis and metastasis of cancer. We found that the expression levels of Mucin-1 (MUC1) and YBX1 were positively correlated in lung adenocarcinoma cells and lung adenocarcinoma tissue. Our retrospective cohort study of 176 lung adenocarcinoma patients after surgery showed that low expression of both YBX1 and MUC1 was an independent predictor of the prognosis and recurrence of lung adenocarcinoma. In lung adenocarcinoma cells, the silencing/overexpression of YBX1 caused a simultaneous change in MUC1, and MUC1 overexpression partially reversed the decreased tumor cell migration, aggressiveness, and stemness caused by YBX1 silencing. Moreover, chromatin immunoprecipitation (ChIP) and dual-luciferase reporter assays proved that MUC1 was the downstream target of YBX1 and that YBX1 bound to the -1480~-1476 position in the promoter region of MUC1 to regulate its transcription. Furthermore, in mouse xenograft models and a lung cancer metastasis model, MUC1, which is downstream of YBX1, partially reversed the decreased number and size of tumors caused by YBX1 silencing. In conclusion, our findings indicated a novel mechanism by which YBX1 promotes the stemness and metastasis of lung adenocarcinoma by targeting MUC1 and provided a combination approach for diagnosis different from traditional single tumor biomarkers to predict patient prognosis and provide clinical treatment targets.

## Introduction

Over the past three decades, lung cancer has become the leading cause of cancer-related deaths worldwide (1, 2). Non-small cell lung cancer (NSCLC) accounts for approximately 85% of lung cancer cases, and adenocarcinoma is the most common subtype (3). Unfortunately, owing to early metastasis and recurrence, the actual prognosis of lung adenocarcinoma has not significantly improved to date (4, 5).

Two important characteristics of cancer stem cells (CSCs) are their ability to self-renew to drive tumorigenesis, and their potential to form tumor heterogeneity due to multidirectional differentiation (6). Accumulating evidence demonstrates that tumorigenesis at metastatic sites and tumor recurrence following treatment occur to CSCs (7, 8). The invasion and migration of tumor cells also related to the malignant behavior of cancer (9, 10). Through epithelial-mesenchymal transition (EMT), epithelial cells lose their polarity, connection with the basement membrane and other epithelial phenotypes, resulting in increased migration and invasion abilities, antiapoptosis activity, extracellular matrix degradation and interstitial phenotypes (11, 12). Consequently, understanding the molecular mechanism underlying the stem-like properties and metastasis of cancer is necessary for improving the prognosis and recurrence of lung cancer.

Mucin-1 (MUC1), a high molecular weight glycoprotein, has become a potential tumor biomarker used in tumor diagnosis and biological treatment due to its abnormal expression in various tumor tissues (13–17). MUC1 can affect E-cadherin and β-catenin expression and interact with E-selectin, making tumor cells easily adhere to vascular endothelial cells and cross vascular walls, to facilitate the metastasis of tumor cells (18–20). MUC1 gene expression is mainly regulated at the transcriptional level (15). This regulation is achieved by the interaction between cis-acting elements on the MUC1 promoter and transcription factors in cells. The MUC1 promoter region contains two Sp1, one Spa, and one E-box (E-MUC1) binding sites (21–23). Interestingly, we identified two potential YBX1-binding sites in the MUC1 promoter (-1743 to -1739 and -1480 to -1476) (24–26). Y-box-binding protein 1 (YBX1) is a multifunctional transcription/translation factor that can bind to a target promoter or enhancer through the Y-box motif, a CCAAT box (or an ATTGG inverted box) (25, 27, 28). A series of downstream target genes of YBX1 are oncogenes that are involved in malignant growth, chemoresistance, metastasis, tumor angiogenesis, and stem-like properties (24, 29–32). Moreover, we found that high YBX1 expression in lung adenocarcinoma is associated with poor prognosis and metastasis in patients (33, 34).

In this article, we aimed to study the relationship between two classic tumor markers, their significance for the prognosis and recurrence of lung adenocarcinoma, and their potential role in regulating the stem-like characteristics and metastasis of lung adenocarcinoma. We hope to provide a combination approach for diagnosis that is different from traditional single tumor biomarkers to predict patient prognosis and offer potential targets for clinical treatment.

## Materials and Methods

### Cell Lines and Cell Culture

Human lung adenocarcinoma cell lines (A549, NCI-H1299, NCI-H322, HCC827, and NCI-H358), one normal human embryonic lung fibroblast cell line (HLF), and 293T cells were purchased from American Type Culture Collection (Manassas, VA, USA). All the cells were cultured in the medium recommended by ATCC with 10% fetal bovine serum (Gibco, Australia) and maintained at 37°C in a humidified 5% CO_2_ incubator.

### Western Blot Analysis

The collected cells or tissues were lysed with RIPA lysis buffer (Beyotime, China, Cat#: P0013B), and protein quantification was performed using a BCA protein assay kit (Thermo Fisher Scientific, USA, Cat#: 23225). Equal amounts of proteins from cell and tissue lysate were subjected to electrophoresis in 10-12% SDS-PAGE and transferred to polyvinylidene difluoride (PVDF) membranes. The membranes were blocked with 8% skim milk and then incubated at 4°C overnight with the indicated the specific antibodies (diluted appropriately according to the instructions), followed by incubation with the appropriate HRP-conjugated secondary antibodies. The target bands were detected with an electrochemiluminescence (ECL) kit (Abbkine, China, Cat#: K22020). The primary antibodies against YBX1 (sc-398340), MMP-9 (sc-21733), MMP-2 (sc-13594), β-catenin (sc-7963), Snail (sc-271977), and Slug (sc-166476) were purchased from Santa Cruz Biotechnology (Santa Cruz, CA, USA). The antibodies against MUC1 (#14161), β-catenin (8480S), Vimentin (#5741), Oct-4 (2750S), were purchased from Cell Signaling Technology (Beverly, MA, USA). The antibodies against GAPDH (10494-1-AP), E-cadherin (20874-1-AP), N-cadherin (22018-1-AP), CD133 (14553-1-AP), MUC1 (19976-1-AP), and CD44 (15675-1-AP) and the goat-anti-rabbit IgG conjugated to horseradish peroxidase (HRP) (HAF008) and goat-anti-mouse IgG conjugated to HRP (HAF007) were purchased from Proteintech (Wuhan, China).

### Reverse Transcription-Polymerase Chain Reaction

Total RNA was extracted by using an RNAiso Plus kit (TaKaRa, China, Cat#: D9108A), which was used to generate cDNA by TransScript^®^ One-Step gDNA Removal and cDNA Synthesis SuperMix (TransGen, China, Cat#: AT311). RT-PCR was performed following the specification of 2×Taq Master Mix (Dye Plus) (Vazyme, China, Cat#: P112). The PCR primer sequences were as follows: YBX1 forward 5′-TGCAGCA GACCGTA ACCATT-3′, reverse: 5′-TGGATCGGCTGCTTTTGTC-3′; MUC1 forward 5′-CAGTGCTTACAGTTGTTACGGG-3′, reverse 5′-CTCAGTAGAGCTGGGCACTG-3′; GAPDH forward: 5′-AATCCCATCACCATCTTCC-3′, reverse 5′-CATCACGCCACAGTTTCC-3′.

### Plasmid Vector and Small-Interfering RNA

The YBX1 overexpression vector (pcDNA3.1-YBX1) and control vector (pcDNA3.1-vector) plasmids were designed and synthesized by Cyagen (Cyagen Biosciences Inc., China). The shRNA expression vector for silencing YBX-1 and the negative control vector were generated by our laboratory: (pGPU6/GFP/Neo-YBX1-homo-746 with the target sequence GGTTCCCACCTT ACTACAT; pGPU6/GFP/Neo-YBX1-homo-326 with the target sequence AGAAGGTCATCGCAACGAA) and the negative control vector (pGPU6/GFP/Neo-shNC) containing the selectable marker GFP. Full-length human MUC1 gene (GenBank: NM_002456) was amplified and cloned into the pcDNA3.1(+) expression vector (Sangon Biotech, China). The constructed plasmid (pcDNA3.1-MUC1) was verified by DNA sequencing. The promoter (- 1874 to + 72) of MUC1 was amplified from the genomic DNA of A549 cells and inserted into pGL3-basic (Clontech, USA) to generate the luciferase construct pGL3-MUC1 driven by the MUC1 promoter. According to the manufacturer’s instructions, the ClonExpress II One Step Cloning Kit (Vazyme, China, Cat#: C112-02) was used for the construction of two mutant plasmids with the Y-boxes deleted at their respective sites (binding site 1: - 1860 to - 1856; binding site 2: - 1468 to - 1464). The pRL-TK Renilla control plasmid was purchased from Clontech (USA). Invitrogen™ Lipofectamine™ 3000 Transfection Reagent (Thermo Fisher, USA) was used for cell transfection.

The primers from CE Design V1.04 for the Luciferase plasmids construction are as below:

MUC1-Full-F-cgagctcttacgcgtgctagcgcgctgacgtcagatgtccc

MUC1-Full-R-cagtaccggaatgccaagcttggtggtggtgaaatgggtgg

MUC1-M1-F-tgctgatctgatgcgctcccgtgcctcgccgaagtgttcccgtgcctcgccgaagt

MUC1-M1-R-agcgcatcagatcagcaggcagcgcatcagatcagcaggc

MUC1-M2-F-tacctgtccaccactctgctccccaaaggataccactctgctccccaaagg

MUC1-M2-R-cagagtggtggacaggtaggcacgtagcgggacaggtaggcacgtagcg

### Immunofluorescence and Confocal Analysis

Cells were cultured on coverslips in culture plates. The coverslip was removed and washed with PBS (HyClone, Logan, UT, USA), fixed with 4% paraformaldehyde for 30 mins, permeabilized with 0.2% Triton X-100 for 3 min and then blocked with 10% BSA (Gibco, Australia) for 30 mins. After that, the coverslips were incubated with primary antibodies (1:200) against YBX1 and MUC1 at 4°C overnight in a wet box, and secondary fluorescent antibodies were added and incubated at room temperature in a wet box in the dark for 1 hour. The cell nuclei were stained with DAPI for 3 min. After washing 5 times with PBS for 10 mins each, the location and expression of the YBX1 and MUC1 protein in the cells were observed by a confocal microscope (Leica, Germany, Cat#: DM 14000B).

### Patients and Specimens

One hundred seventy-six consecutive patients with lung adenocarcinoma (median age: 67years; range 47 from 85 years) who underwent radical surgery of the primary tumor and systematic nodal dissection without any adjuvant therapy at the Thoracic Surgery of the First Affiliated Hospital of Dalian Medical University from January 2008 to December 2010 were included. Patient follow-up was performed according to our previous study, and the final follow-up time was January 2014 (median follow-up: 1213days). The research was approved by the Medical Ethical Committees of the First Affiliated Hospital of Dalian Medical University. All the patients signed written informed consent and agreed that their tissue samples could be used for clinical research, but not for commercial use. The ethics committee procedure number is PJ-KS-KY-2021-235.

### Immunohistochemical Staining

All the specimens were obtained from patient’s resected primary lesions, or mouse models, fixed with formalin, and embedded in paraffin, and 4-μm serial sections were prepared for later use. Immunohistochemical staining (IHC) was performed according to the manufacturer’s manual of the 3, 3′-diaminobenzidine (DAB) Kit (ZSGB-Bio, China, Cat#: sp-9000). Then the slides were observed and photographed with an upright microscope.

The immunostaining of patient specimens was evaluated by three pulmonary pathologists, including a senior pathologist if necessary, using a blind protocol design. For each specimen, the total score of YBX1 and MUC1 expression was estimated as follows: expression intensity (negative staining: 0 points; weak staining: 1points; moderate staining: 2 points; and intense staining: 3 points) multiplied by numbers of stained cells (≤25% of the cells were positive: 1 point; 26-50% of the cells were positive: 2 point; 51-75% of the cells were positive: 3 point; >75% of the cells were positive: 4 point). When a sample scored ≥6 points, we defined the sample as having high expression; otherwise, the sample was defined as having low expression. The positive controls of YBX1 and MUC1 were obtained from the Human Protein Atlas website (http://www.proteinatlas.org).

### The Actinomycin D Transcription Repression Assay

The equal cells are grown in culture plates. After overexpressing YBX1, the Actinomycin D (MCE, USA, Cat#: HY-17559) with recommended concentration (2 μg/ml) was added to the plates (including the Control and Overexpression groups) at the same time. Cells were collected after 0, 0.5, 1, and 2 hours, respectively, and then the expression of MUC1 mRNA at different times was detected according to the method in 2.3.

### Dual-Luciferase Reporter Assay

When (1×10^5^) cells in a 6-well plate were attached, we transfected MUC1 promoter-driven luciferase constructs or the control (pGL3-Basic) luciferase plasmid and the pRL-TK Renilla control plasmid using Lipofectamine 3000 (Thermo Fisher, USA). After 48 hours, cell samples were harvested. The firefly and Renilla luciferase activities were measured using a dual-luciferase assay kit (Promega, USA, Cat#: E1910). The relative firefly luciferase data of the samples were normalized by measuring the Renilla luciferase activity (pRL-TK Renilla control plasmid).

### Chromatin Immunoprecipitation Assay

Genomic DNA fragments were collected from lung adenocarcinoma cells lysed by ultrasound. The remaining procedures followed the manufacturer’s instructions for the ChIP IT Express kit (Active Motif, Rixensart, Belgium). After specific fragments were immunoprecipitated with a specific YBX1 antibody, specific primers were used to amplify the MUC1 promoter sequence. The primers used for the amplification of the immunoprecipitated DNA fragments were as follows: Site-1 forward 5′-GTGGTCTCTCGCCTGCTGAT-3′, reverse 5′-GGAAGCAGTTCCGCCTGTA-3′; Site-2 forward 5′-GATAGCTTCCTCCCCTCGTG-3′, reverse: 5′-CTATCCTTTGGGGAGCAGAGTG-3′).

### Wound-Healing Assay

Cell migration abilities can be observed and compared by the scratch test. When the cells were almost confluent, wound gaps were generated by gently scraping with a 200-μl pipette tip. Then, the cells were cultured in 1% FBS medium to minimize the effects of proliferation. The spacing of the gap was photographed by an inverted microscope at 0 hour and 24 hours. The percent cell migration distance was calculated as follows: (1-mean remaining breadth/mean initial breadth) ×100%.

### Migration and Invasion Assays

The same number of cells (5×10^3^ for migration assay, 1×10^4^ for invasion assay) were seeded on polycarbonate Transwell filters (Corning, CA, USA, Cat#:3422). Matrigel (BD, Franklin Lakes, NJ, USA, Cat#:356234) was used in invasion assay in 24-well plates and incubated at 37°C (24 hours for migration assay, 36 hours for invasion assay). The cells penetrating the membrane were fixed in 4% paraformaldehyde for 10 min, stained with 0.5% crystal violet and counted (Olympus, Japan, Cat#: IX71).

### Sphere Formation Assay

The same number of cells (1000) were seeded into ultralow attachment culture dishes and cultured in DMEM/F12 serum-free medium (Invitrogen) supplemented with 2% B27 (Thermo Fisher, USA), 1% N2 (Thermo Fisher, USA), 20 ng/ml epidermal growth factor (Sigma-Aldrich, USA) and 10 ng/ml basic fibroblast growth factor (BD, Franklin Lakes, NJ, USA), for 10 days. The cell spheres were observed and photographed by an inverted microscope, and the Image-Pro Plus software was used to measure the longest diameter of spheres by the contrast scale in the pictures.

### ALDH Activity Detection

Cells were gently harvested, and the concentration was adjusted to 5×10^5^/ml. The instructions of the ALDEFLUOR™ fluorescent reagent system (STEMCELL, Canada, Cat#: #01700) were followed to carefully prepare the samples. The activity of the enzyme aldehyde dehydrogenase (ALDH) in the cells was detected by flow cytometry.

### Mouse Xenograft Model and Lung Cancer Metastasis Model

Twenty-four male BALB/c athymic nude mice (4-6 weeks old) were randomly divided into four groups. A549 cells (2×10^6^) were subcutaneously injected into the ipsilateral armpit of each mouse. Seven days after injection, the tumor volume (v = (width^2^ × length)/2) and body weight were recorded with a Vernier caliper every 3 days. Tumor specimens were fixed in formalin and then embedded in paraffin; some of them were preserved in liquid nitrogen for other analysis. In the mouse lung cancer metastasis model assay, 12 male BALB/c athymic nude mice (4-6 weeks old) were injected *via* the tail vein with A549 cells (5×10^5^). Forty-five days after injection, the distribution of metastasis in the lungs of the mice was detected by *in vivo* imaging. After saving a small amount of tissue for Western blot analysis, the whole lung was fixed in formalin for paraffin embedding and sectioning, and HE staining was used to observe the size and shape of the metastases. The ethics committee procedure number is AEE19015.

### Statistical Analysis

Student’s t-test or analysis of variance (ANOVA) was used to compare the values of the *in vitro* and *in vivo* test and control samples. The associations between the YBX1 or MUC1 expression levels and categorical variables were compared by the Pearson chi-square test. Survival curves were calculated using the Kaplan-Meier method. The log-rank test was used to analyze OS and DFS between different clinicopathological factors in lung adenocarcinoma. Multivariate analysis was performed using Cox regression analysis. The data were analyzed by SPSS 25 software (SPSS Inc., Chicago, IL, USA). Values of p<0.05 were considered statistically significant.

## Results

### Expression Levels of YBX1 and MUC1 Were Correlated in Lung Adenocarcinoma Cells and Tissues

**Figure 1:**
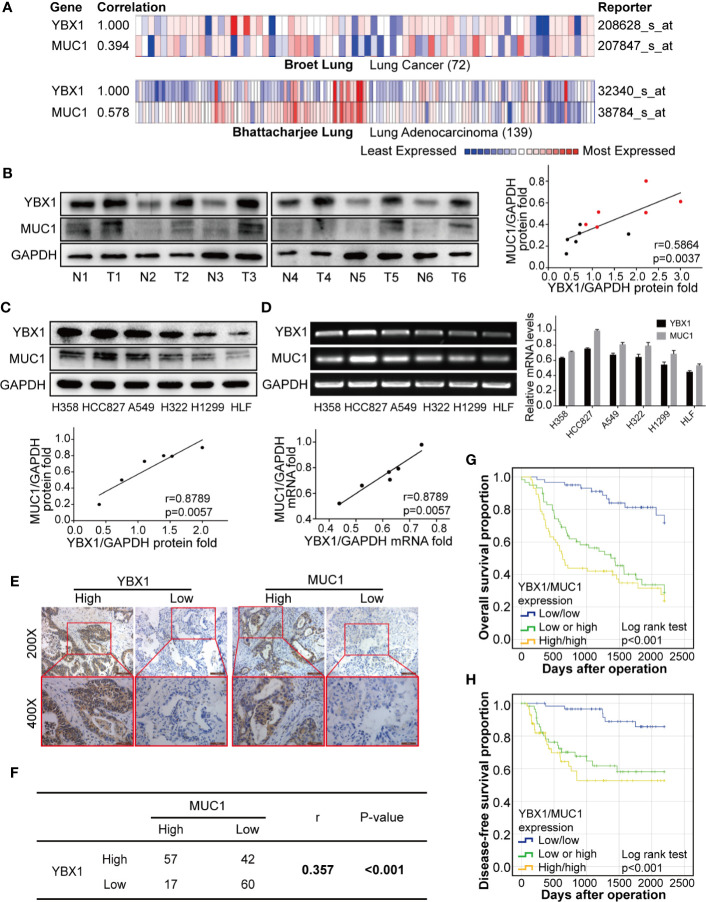
YBX1 and MUC1 expression was the highly correlated and affected prognosis. **(A)** YBX1 and MUC1 mRNA expression were based on the Broet Lung and Bhattacharjee Lung datasets from the Oncomine database (p<0.05, p<0.05, respectively). **(B)** Western blot analysis of YBX1 and MUC1 expression in 6 pairs of lung adenocarcinoma (T) and adjacent tissues (N) (r = 0.5864, P < 0.0037, Red dot: T, Black dot: N). **(C)** Western blot analysis of YBX1 and MUC1 expression in HLF and 6 lung adenocarcinoma cell lines (r = 0.8789, P < 0.0057). **(D)** RT-PCR analysis of YBX1 and MUC1 expression in HLF and lung adenocarcinoma cell lines (r = 0.9306, P < 0.0018). **(E)** IHC analysis of YBX1 and MUC1 expression in lung adenocarcinoma. Original magnification 200× and 400× in the inset. **(F)** YBX1 and MUC1 expression was positive correlated in 176 lung adenocarcinoma specimens (r = 0.357, P < 0.001). **(G)** The OS curves of YBX1/MUC1 expression in 176 patients with lung adenocarcinoma (p <0.001). **(H)** The DFS curves of YBX1/MUC1 expression in 176 patients (p <0.001). The data are presented as mean ± SD of three independent tests.

To analyze the expression of YBX1 and MUC1 in human lung adenocarcinoma, we first analyzed the Oncomine database and found that there was a significant correlation between the YBX1 and MUC1 mRNA levels in lung cancer patient tissues (r=0.394; p<0.05), especially lung adenocarcinoma tissues(r=0.578; p<0.05; **Figure 1A**) (35, 36). Then we detected the expression of YBX1 and MUC1 in the cancer tissues and adjacent tissues of 6 lung adenocarcinoma patients, and we observed significantly high expression of both in the tumors. Quantitative analysis showed that there was a significant correlation (**Figure 1B**, r=0.5864; p=0.0037). In addition, we analyzed the expression of YBX1 and MUC1 in HLF cell and five lung adenocarcinoma cell lines (H358, HCC827, A549, H322, H1299) (**Figures 1C, D**), and they were significantly correlated at both the protein (**Figure 1C**, r=0.8789, p=0.0057) and mRNA (**Figure 1D**, r=0.9306, p=0.0018) levels.

### YBX1 and MUC1 Predicted Lung Adenocarcinoma Patient Prognosis and Recurrence

These data were based on a retrospective cohort study of 176 patients with lung adenocarcinoma after surgery. The basic clinicopathological parameters of all the enrolled patients are summarized in **Table 1**. The expression of YBX1, as detected by IHC was usually found in the cytoplasm, while MUC1 appeared more frequently in the nucleus and cell membrane (**Figure 1E**). Among all 176 patients, 99(56.3%) and 74 (42.0%) patients showed high expression of YBX1 and MUC1, respectively.

**Table 1:** Clinicopathologic characteristics in 176 patients with completely resected lung adenocarcinoma.

Characteristics	Number (%)
Sex	
male	103 (58.2)
female	73 (41.8)
Age	
≤67y	94 (53.4)
>67y	82 (46.6)
Differentiation	
well	61 (34.7)
moderate	84 (47.7)
poor	31 (17.6)
T state	
T1	80 (45.4)
T2	60 (34.1)
T3	13 (7.4)
T4	23 (13.1)
N state	
N0	126 (71.6)
N1	13 (7.4)
N2	35 (19.9)
N3	2 (1.1)
M state	
M0	170 (96.6)
M1	6 (3.4)
TNM stage	
I	115 (65.3)
II	9 (5.1)
IIIA	22 (12.5)
IIIB/IV	30 (17.1)
YBX1 expression	
High	99 (56.3)
low	77 (43.7)
MUC1 expression	
High	74 (42.0)
low	102 (58.0)

The relationship between clinicopathologic characteristics and YBX1 and MUC1 expression in these patients was summarized in **Table 2**. The expression of YBX1 was significantly correlated with differentiation, T state, N state, and TNM stage (p=0.002, p<0.001, p=0.001, p<0.001, respectively). Additionally, MUC1 expression in patients was related to differentiation, N state, and TNM stage (p=0.049, p=0.025, p=0.007, respectively). Furthermore, we found a certain positive correlation between the expression levels of YBX1 and MUC1 (r=0.357; p<0.001) according to the IHC results of 176 lung adenocarcinoma specimens (**Figure 1F**).

**Table 2:** Genomic and epigenomic differences between nasopharyngeal carcinomas (NPCs), lymphoepithelioma-like carcinomas (LELCs) and EBV-associated gastric carcinomas (EBVaGCs).

Characteristics	YBX1		P-value	MUC1		P-value
	High (%)	Low		High (%)	Low	
Over all	99 (56.3)	77		74 (42.0)	102	
Sex			0.210			0.38
male	62 (60.2)	41		50 (48.5)	53	
female	37 (50.7)	36		24 (32.9)	49	
Age			0.381			0.440
≤67y	50 (53.2)	44		37 (39.4)	57	
>67y	49 (59.8)	33		37 (45.1)	45	
Differentiation			**0.002**			**0.049**
well	24 (39.3)	37		18 (29.5)	43	
moderate	52 (61.9)	32		41 (48.8)	43	
poor	23 (74.2)	8		15 (48.4)	16	
T state			**<0.001**			0.103
T1	29 (36.3)	51		30 (37.5)	50	
T2	40 (66.7)	20		23 (38.3)	37	
T3	9 (69.2)	4		6 (46.2)	7	
T4	21 (91.3)	2		15 (65.2)	8	
N state			**0.001**			**0.025**
N0	59 (46.8)	67		44 (34.9)	82	
N1	12 (92.3)	1		8 (61.5)	5	
N2	27 (77.1)	8		21 (60.0)	14	
N3	1 (50.0)	1		1 (50.0)	1	
M state			0.30			0.499
M0	93 (54.7)	77		71 (41.8)	99	
M1	6 (100.0)	0		3 (50.0)	3	
TNM stage			**<0.001**			**0.007**
I	51 (44.3)	64		40 (34.8)	75	
II	5 (55.6)	4		2 (22.2)	7	
IIIA	16 (72.7)	6		13 (59.1)	9	
IIIB/IV	27 (90.0)	3		19 (63.3)	11	

Statistical significance was evaluated using the chi-square test. Differences were considered to be statistically significant for P values of < 0.05 which are shown in bold.

Univariate analysis by Kaplan-Meier analysis showed that male sex, poor differentiation, advanced TNM stage, and high coexpression of YBX1/MUC1 (p=0.027, p<0.001, p<0.001, p<0.001, respectively) were associated with poorer overall survival (OS). We also found good differentiation, low TNM stage, and low coexpression of YBX1/MUC1 (p=0.007, p=0.013, p<0.001, respectively) and lower recurrence (**Table 3**, **Figures 1G, H**). The multivariate Cox regression analysis indicated us that good differentiation (hazard ratio, [HR] =0.386; 95% confidence interval [CI]: 0.182-0.816; p=0.013), N0 state (HR=0.121, 95% CI: 0.023-0.629, p=0.012), IIIA stage (HR=0.327, 95% CI: 0.160-1.669, p=0.002) and low YBX1/MUC1 coexpression (HR=0.241; 95% CI: 0.119-0.441; p<0.001) were independent prognostic factors of overall survival in lung adenocarcinoma (**Table 4**). Moreover, the low coexpression of YBX1/MUC1 was an independent protective factor of free-disease survival ([DFS]; HR=0.160; 95% CI: 0.064-0.398; p<0.001), and these patients had a lower recurrence (**Table 5**). In conclusion, the low coexpression of YBX1/MUC1 showed vital prognostic significance in the prognosis and recurrence of lung adenocarcinoma.

**Table 3:** Univariate analysis of five-year overall survival and disease-free survival on different clinicopathological factors using Kaplan–Meier method.

Risk factor	5-OS (%)	Log-rank test	5-DFS (%)	Log-rank test
		(P-value)		(P-value)
Sex		**0.027**		0.322
male	44.2		65.7	
female	58.6		69.3	
Age		0.948		0.831
≤67y	46.7		66.3	
>67y	54.3		67.9	
Differentiation		**<0.001**		**0.007**
well	74.0		78.2	
moderate	37.9		61.0	
poor	38.7		57.2	
T state		**<0.001**		**0.024**
T1	70.3		77.0	
T2	46.4		56.0	
T3	23.1		55.9	
T4	8.7		76.6	
N state		**<0.001**		**0.003**
N0	64.7		72.1	
N1	23.1		69.2	
N2	15.0		43.5	
N3	0.0		0.0	
M state		**0.001**		0.197
M0	51.7		67.5	
M1	0.0		66.7	
TNM stage		**<0.001**		**0.013**
I	66.8		71.8	
II	44.4		66.7	
IIIA	27.6		48.0	
IIIB/IV	7.0		49.3	
MUC1/YBX1 expression		**<0.001**		**<0.001**
Low/low	87.3		85.8	
Low or high	37.0		58.1	
High/high	31.6		52.7	

Statistical significance was evaluated using the log-rank test. Differences were considered to be statistically significant for P values of < 0.05 which are shown in bold.

**Table 4:** Multivariate analysis of overall survival using Cox regression.

Risk factor	Multivariate analysis		P-value
	HR	95%CI	
Sex			
male	1.229	0.754-2.004	0.408
female	1		
Differentiation			
well	0.386	0.182-0.816	**0.013**
moderate	0.905	0.492-1.665	0.748
poor	1		
T state			
T1	0.387	0.100-1.502	0.170
T2	0.605	0.171-2.139	0.435
T3	1.033	0.332-3.214	0.955
T4	1		
N state			
N0	0.121	0.023-0.629	**0.012**
N1	0.229	0.042-1.239	0.087
N2	0.499	0.107-2.323	0.375
N3	1		
M state			
M1	1.148	0.128-10.266	0.902
M0	1		
TNM stage			
I	0.561	0.216-1.459	0.236
II	0.856	0.300-2.441	0.771
IIIA	0.327	0.160-1.669	**0.002**
IIIB/IV	1		
MUC1/YBX1 expression			
Low/low	0.241	0.119-0.441	**<0.001**
Low or high	0.951	0.588-1.537	0.836
High/high	1		

Statistical significance was evaluated using the Cox regression test. Differences were considered to be statistically significant for P values of <0.05 which are shown in bold.

**Table 5:** Multivariate analysis of disease-free survival using Cox regression.

Risk factor	Multivariate analysis		P-value
	HR	95%CI	
Differentiation			
well	0.597	0.244-1.460	0.258
moderate	0.893	0.422-1.889	0.768
poor	1		
T state			
T1	0.866	0.287-2.611	0.798
T2	1.674	0.603-4.649	0.323
T3	1.386	0.359-5.357	0.636
T4	1		
N state			
N0	0.222	0.029-1.695	0.147
N1	0.214	0.022-2.122	0.188
N2	0.447	0.057-3.524	0.445
N3	1		
TNM stage			
I	1.093	0.111-10.811	0.937
II	0.929	0.098-8.775	0.949
IIIA	0.523	0.106-2.587	0.427
IIIB/IV	1		
MUC1/YBX1			
Low/low	0.160	0.064-0.398	**<0.001**
Low or high	0.776	0.420-1.432	0.417
High/high	1		

Statistical significance was evaluated using the Cox regression test. Differences were considered to be statistically significant for P values of <0.05 which are shown in bold.

### YBX1 May Regulate the Expression of MUC1 Through Transcription

**Figure 2:**
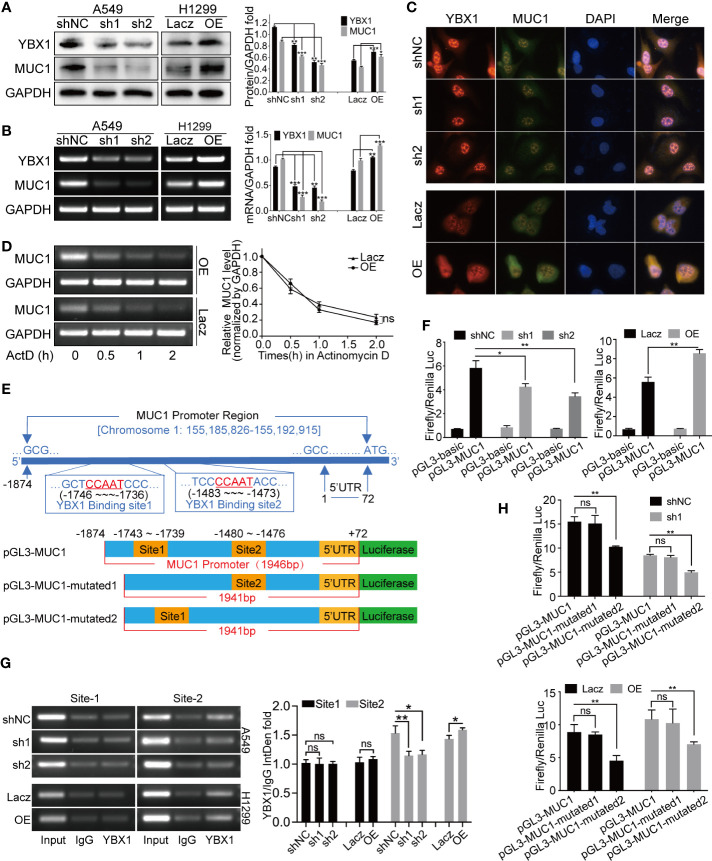
YBX1 affected MUC1 expression through transcriptional regulation. **(A)** Western blot analysis of YBX1 and MUC1 expression in A549 cells with YBX1 silence (sh1 or sh2), and in H1299 cells with YBX1 overexpression (OE). **(B)** RT-PCR analysis of YBX1 and MUC1 in A549 cells with YBX1 silence, and in H1299 cells with YBX1 overexpression. **(C)** The YBX1 and MUC1 expression and the YBX1 nuclear translocation were detected by immunofluorescence assay, after silencing YBX1 in A549 or overexpression of YBX1 in H1299. **(D)** Actinomycin-D transcription repression assay showed that the stability of MUC1 mRNA did not change significantly after YBX1 overexpression. **(E)** The location of two putative YBX1 binding sites in the MUC1 promoter region, and three plasmids required for the dual-luciferase reporter assay. **(F)** The dual-luciferase assay showed that the promoter region of MUC1 was positively activated by YBX1. **(G)** The ChIP assay found the binding of YBX1 was at putative site 2, and the activation was affected by YBX1 expression. **(H)** The dual-luciferase activity was significantly reduced after mutating site 2, compared with mutating site 1, and affected by YBX1 expression. The data are presented as mean ± SD of three independent tests. *P < 0.05, **P < 0.01, ***P < 0.001. ns, non-significance.

We selected A549 cells with relatively high expressing for YBX1 silencing and H1299 cells with relatively low expression for YBX1 overexpression. Synchronous changes in YBX1 and MUC1 were observed at both the protein and mRNA levels (**Figures 2A, B**). Fluorescence colocalization revealed that YBX1 was expressed in the cytoplasm and translocated in the nucleus, and MUC1 was mainly expressed in the cytoplasm. After YBX1 silencing, the expression and nuclear translocation of YBX1 and MUC1 expression in A549 cells were reduced. After overexpression, the expression and nuclear translocation of YBX1 and expression of MUC1 in H1299 cells increased (**Figure 2C**). We designed an actinomycin D (Act-D) transcription repression assay to obtain the MUC1 mRNA decay curves of H1299 cells in the YBX1 overexpression group and control group. The data showed that YBX1 overexpression did not significantly delay MUC1 mRNA degradation (**Figure 2D**), so YBX1 may be a transcriptional regulator of MUC1.

### YBX1 Bound to and Regulated the Promoter Region of MUC1

We designed two putative YBX1 binding site primers that were needed in the ChIP assay (**Figure 2E**) and three plasmids that were needed in the dual-luciferase reporter assay (**Figure 2E**). In **Figure 2F**, the activation of MUC1 promoter by YBX1 was observed. YBX1 silencing in A549 cells resulted in a significant decrease in this activation, and YBX1 overexpression in H1299 cells increased this activation. Subsequently, two YBX1 binding primers were used to amplify the genomic fragment of MUC1 promoter that binds to YBX1, and it was found that the binding occurred at site 2. The binding was weakened/intensified with YBX1 silencing/overexpression (**Figure 2G**). By comparing the activation of the three luciferase plasmids, it was found that site 2 was essential for YBX1 to bind and activate the MUC1 promoter region, and this binding and activation was related to YBX1 expression (**Figure 2H**).

### YBX1 Enhanced Lung Cancer Cells Migration and Invasion

**Figure 3:**
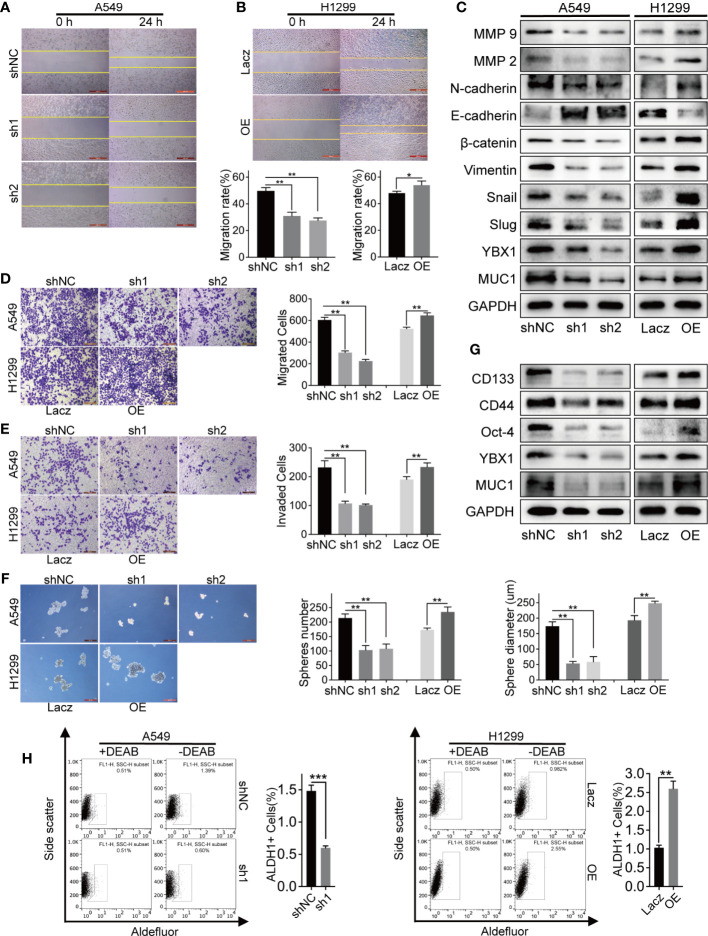
The expression levels of YBX1 affected lung adenocarcinoma cells migration, invasion, and stemness. **(A, B)** The wound healing assay analyzed cell migration in A549 and H1299 cells, and YBX1 expression affected the migration rate. **(C)** YBX1 expression affected the EMT signaling and MMPs family. **(D)** The transwell assay without matrigel analyzed cell migration in A549 and H1299, and YBX1 expression affected the migrated cells number. **(E)** The transwell assay with matrigel analyzed cell invasion in A549 and H1299, and YBX1 expression affected the invaded cells number. **(F)** The sphere formation analyzed cancer cell stemness in A549 and H1299 cells. The number and diameter of the spheres were affected by YBX1 expression. **(G)** The markers related to stemness were detected by western blot, including CD44, CD133, Oct-4. **(H)** ALDH1 positive population analysis revealed that decreased expression of YBX1 reduced the ALDH1+ cell proportions, whereas overexpression of YBX1 increased the proportion. The data are presented as mean ± SD of three independent tests. *P < 0.05, **P < 0.01, ***P < 0.001.

To study the functional significance of YBX1 in lung adenocarcinoma, we first examined the migration and invasion capabilities, which are closely related to cancer metastasis and recurrence. In the 24-hour wound healing assay, we found that the silencing/overexpression of the upstream gene YBX1 played a vital role in weakening/enhancing the migration ability of lung adenocarcinoma cells (**Figures 3A, B**). In the Transwell systems used to detect either migration or invasion, overexpression of YBX1 in H1299 cells increased the number of cells passing through the membrane, while silencing of YBX1 in A549 cells decreased migration or invasion (**Figures 3D, E**). The expression of the EMT markers and MMP family members has the same variance tendency as that of YBX1 in A549 and H1299 cells, except that E-cadherin had the opposite trend (**Figure 3C**). All these results show that YBX1 is involved in the control of migration and invasion of lung cancer cells.

### YBX1 Promoted Lung Cancer Stem-Like Properties

We verified the role of YBX1 in promoting self-renewal ability, a critical trait of CSCs, through sphere formation and ALDH1-positive (ALDH1+) cell sorting assays. YBX1 silencing weakened the sphere formation efficiency of A549 cells, as shown by decreased sphere number and diameter. In contrast, the efficiency of H1299 cells increased significantly after YBX1 overexpression (**Figure 3F**). Consistent with sphere formation, YBX1 silencing significantly reduced the ALDH1+ population in A549 cells, whereas YBX1 overexpression increased the ALDH1+ population in H1299 cells (**Figure 3H**). We detected CD133, CD44, and Oct-4, which are vital markers of lung cancer stem cells, by Western blot. In A549 and H1299 cells, the expression decreased/increased with YBX1 silencing/overexpression (**Figure 3G**). These results indicated that YBX1 has collectively been demonstrated to be pivotal in maintaining the stem-like properties of lung cancer stem cells.

### MUC1 Partially Rescued the Migration and Invasion That Were Decreased by YBX1 Silencing

**Figure 4:**
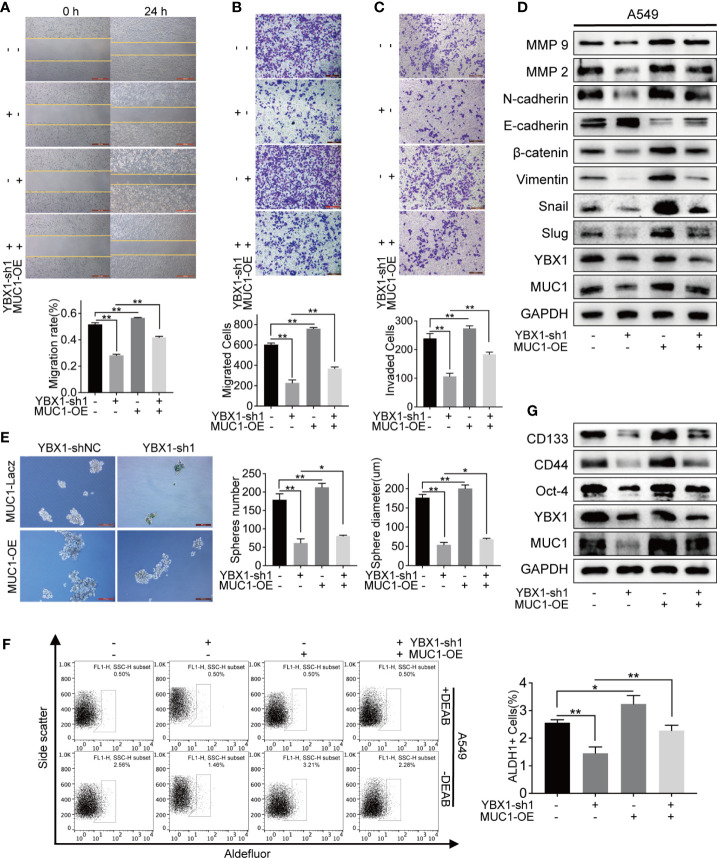
MUC1 overexpression partly rescued the down-regulation of metastasis and stemness caused by YBX1 silencing. **(A)** Cell migration was analyzed by wound healing assay in A549, and the migration rate was partly rescued by MUC1 overexpression. **(B)** Cell migration was analyzed by transwell assay without matrigel, and the migrated cells were partly rescued by MUC1 overexpression. **(C)** Cell invasion was analyzed by transwell assay with matrigel, and the invaded cells were partly rescued by MUC1 overexpression. **(D)** MUC1 overexpression partly rescued the regulation of the EMT signaling and MMPs family caused by YBX1 silencing. **(E)** Cancer cell stemness was analyzed by sphere formation in A549. The number and diameter of the spheres were partly rescued by MUC1 overexpression. **(F)** ALDH1 positive population analysis revealed that MUC1 overexpression increased the number of ALDH1+ cells reduced by YBX1 silencing. **(G)** MUC1 overexpression partly rescued the regulation of the markers related to stemness, including CD44, CD133, Oct-4, caused by YBX1 silencing. The data are presented as mean ± SD of three independent tests. *P < 0.05, **P < 0.01.

We overexpressed MUC1 after YBX1 silencing to verify our hypothesis about the relationship between YBX1 and MUC1. In the 24-hour wound healing assay, after MUC1 overexpression, the cell migration distance was significantly increased compared with that in A549 cells in which MUC1 was not overexpressed (**Figure 4A**). In the transwell migration and invasion system, the number of cells passing through the membrane decreased due to the silencing of YBX1, and a definite increase was obtained after MUC1 overexpression (**Figures 4B, C**). Then, we retested the EMT markers and MMP family members previously detected in A549 cells. The downregulated expression due to YBX1 silencing was restored to some extent after MUC1 overexpression, except that E-cadherin had the opposite trend (**Figure 4D**).

### MUC1 Partially Rescued Cancer Stem-Like Properties After YBX1 Silencing

Next, we tested whether MUC1 overexpression could rescue lung cancer stem-like properties after YBX1 silencing. In the sphere formation assay, the sphere formation efficiency in the MUC1 overexpression group was significantly improved compared with that in the YBX1-shNC and YBX1-sh groups, in which MUC1 was not overexpressed in the A549 cells (**Figure 4E**). In the ALDH1+ cell sorting assay, the number of ALDH1+ cells decreased due to YBX1 silencing, and a significant increase was observed after MUC1 overexpression (**Figure 4F**). After MUC1 overexpression, the downregulated expression of CD133, CD44, and Oct-4 due to YBX1 silencing was rescued (**Figure 4G**).

### YBX1 Promotes Lung Cancer Growth by Regulating MUC1 in Xenograft Mouse Models

**Figure 5:**
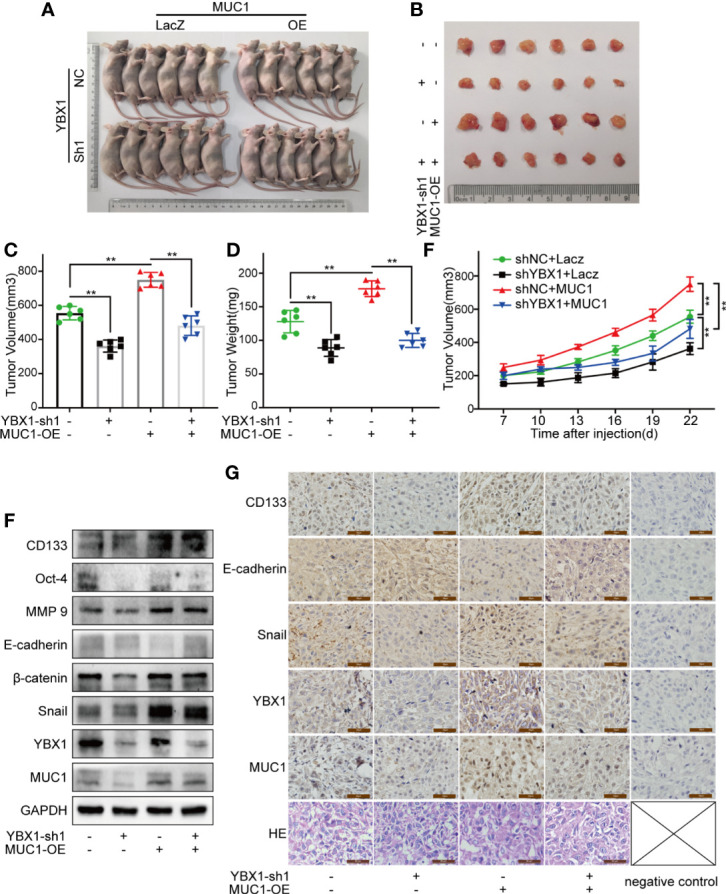
YBX1 promotes tumor growth by upregulating MUC1 in human lung cancer xenograft mouse models. **(A)** The morphology of all mice participating in the experiment was photographed. **(B)** The morphology of tumor xenografts from all mice was photographed. **(C, D)** The tumor volume and weight from the different groups were measured. **(E)** The tumor volume of each mouse was measured at a regular interval of 3 days after 7 days of injection. **(F)** The expression of YBX1, MUC1, CD133, Oct-4, MMP9, E-cadherin, β-catenin, and Snail within xenografts in the different groups were detected by Western blot. **(G)** The expression of YBX1, MUC1, CD133, E-cadherin, and Snail in tumor tissues was detected by IHC staining. The xenograft tissue morphology showed by HE staining. Scale bars= 50μm. The level of significance was indicated by **P < 0.01

To study the effects of YBX1 and MUC1 in xenograft mouse models, we first established A549 cells in which YBX1 was stably silenced or in which YBX1 was silenced and MUC1 was overexpressed. We implanted A549 cells into the armpits of nude mice and then assessed the changes in tumor weight and volume. The results showed that while YBX1 silencing reduced tumor growth, MUC1 overexpression significantly promoted tumor growth (**Figures 5A–E**). Western blot analysis of tumor tissues showed that MUC1 overexpression partially rescued the effect of YBX1 silencing on CD133, Oct-4, MMP9, E-cadherin, β-catenin, and Snail expression (**Figure 5F**). In addition, IHC analysis of tumor sections showed similar results (**Figure 5G**). These *in vivo* data confirmed that YBX1 promoted tumor growth by promoting MUC1 expression.

### YBX1 and MUC1 Affect the Formation of Metastasis in Lung Cancer Metastasis Models

**Figure 6:**
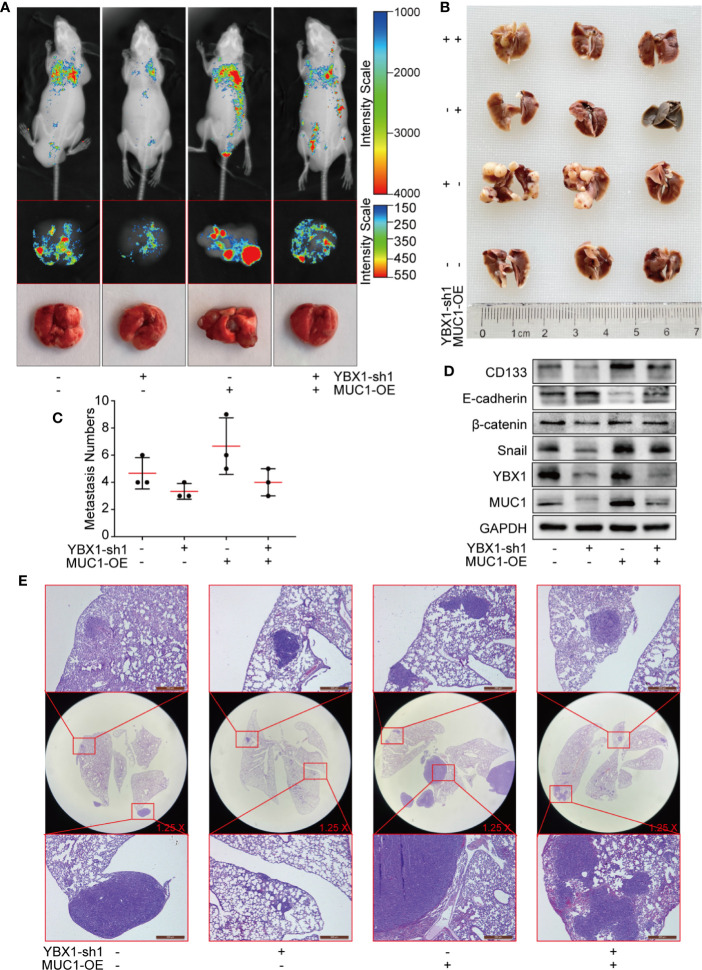
YBX1 promotes tumor metastasis by upregulating MUC1 in lung cancer metastasis models. **(A)** The GFP-labeled A549 from different groups were injected into the tail vein of nude mice, and *in vivo* imaging was performed 45 days later. The lungs are dissected and imaged individually. **(B)** All whole lung was fixed with 4% paraformaldehyde, and the surface of the fixed metastases was white granular. **(C)** The number of metastases in the lungs of each nude mouse was counted. **(D)** The expression of YBX1, MUC1, CD133, E-cadherin, β-catenin, and Snail within xenografts in the different groups was detected by Western blot. **(E)** The lung adenocarcinoma metastasis morphology showed by HE staining. Scale bars= 500μm. N= 3 mice/group. Original magnification of the whole lung: ×1.25.

We established hematological metastasis models by injecting GFP-labeled A549 xenograft mouse model cells into the tail vein of nude mice. As shown (**Figure 6A**), YBX1 silencing and MUC1 overexpression significantly changed the fluorescence intensity observed in *in vivo* imaging. After dissection, the fluorescence imaging of the lungs was similar to that observed during *in vivo* imaging. All whole lungs were fixed with 4% paraformaldehyde and paraffin-embedded sections were obtained after some metastases were saved for protein verification. The surface of the fixed metastases was white and granular (**Figure 6B**). YBX1 silencing and MUC1 overexpression significantly changed the number and size of metastases (**Figures 6B, C**). The Western blot assay of metastases revealed that the overexpression of MUC1 partially restored the changes in CD133, E-cadherin, β-catenin, and snail caused by YBX1 silencing (**Figure 6D**). HE staining of the whole lung sections also verified the effect of YBX1 and MUC1 expression on the size and number of metastases (**Figure 6E**). These data demonstrated that the regulation of MUC1 by YBX1 plays a vital role in the process of blood metastasis.

## Discussion

Lung adenocarcinoma has become one of the most concerning malignancies in recent decades due to its poor prognosis (1, 37). In our study, the N state and TNM stage were strongly related to the 5-year OS of patients with lung adenocarcinoma after surgery (**Table 2**), indicating the pivotal role of early diagnosis and treatment in improving prognosis. The high heterogeneity of lung cancer makes the reliability of traditional single diagnostic tumor markers questionable (38, 39). Therefore, we hope to find a combination approach to diagnosis that is different from traditional single tumor markers to accurately predict patient prognosis, guide actual clinical treatment and study the molecular mechanism to provide clinical therapeutic targets.

As classic tumor biomarkers, YBX1 and MUC1 are significant in the occurrence and development of various tumors (17, 33, 34, 39, 40). In previous studies, YBX1 and MUC1 were crucial in predicting the prognosis and recurrence of lung cancer (33, 40). RNA-seq analysis from Oncomine and TCGA found that YBX1 and MUC1 mRNA expression levels in lung cancer tissues were highly correlated, especially in lung adenocarcinoma (35, 36). We determined a high correlation between the protein and mRNA expression levels of two classic tumor markers in lung adenocarcinoma tissues and cell lines for the first time.

In the retrospective cohort study including 176 lung adenocarcinoma patients after surgery, we found that the YBX1 and MUC1 expression levels were highly correlated in lung adenocarcinoma (**Figure 1F**, r=0.357, P<0.001). Because of lung adenocarcinoma’s high heterogeneity, we did not choose the commercial tissue chip or its related clinical data (41–43). All information was obtained from patients who underwent surgery at our hospital and had complete follow-up records, which avoided the shortcomings of poor representation from small chip tissue and ensured the authenticity and reliability of clinical materials and follow-up records. Univariate analysis (**Table 2**) showed that 5-year OS was related to sex. This may be because all 176 patients were enrolled consecutively, and such a small probability event from the strict criteria for enrollment and exclusion of patients did not affect the authenticity and reliability of the data. Similar to existing research, YBX1 or MUC1, as separate tumor markers, can predict prognosis in lung adenocarcinoma patients. After combining YBX1 and MUC1, the low coexpression group was significantly different from the other groups (**Table 2**, 5-OS: 87.3%, 5-DFS: 85.8%), and this low coexpression was the only protective factor for DFS (**Table 4**). Our research provides a new combined diagnostic tool for the prognosis of lung adenocarcinoma patients. Therefore, we further elaborated on the possible molecular mechanism of YBX1 and MUC1 as a combined diagnostic method.

YBX1 has been considered a therapeutic target for a long time, even the antisense DNA complex of YBX1 has been developed to silence YBX1 in lung cancer cells (44). In previous studies, Y-box sequence, as a cis-acting element, up-regulated ceramide synthase 6 through the transcriptional regulation of YBX1 to promote lamellipodia formation as well as migration activity, and up-regulated TGFBR1 to promote the stemness and EMT of lung cancer cells (45, 46). In the runx3/miR-148a-3p/YBX1 axis, YBX1 was regulated to affect proliferation, invasion, growth, and apoptosis of lung cancer cells, changes slug-1, MMP-2, and MMP-9 expression like our article (47). It can also be directly combined with long-chain non-coding RNA (LINC00312) to induce the migration, invasion, and angiogenesis of lung cancer cells (48, 49). Our composite research includes bioinformatics analysis, retrospective cohort study, mechanisms *in vitro*, and different animal models, which make an important complement to the study of YBX1 affecting growth and metastasis mechanisms in lung cancer.

MUC1 gene expression is mainly regulated at the transcriptional level (15). The MUC1 promoter region contains two Sp1, one Spa, and one E-box (E-MUC1) binding site (21–23). Interestingly, in our further exploration, we found that the MUC1 promoter region had two reverse Y-box sequences, which was the first attempt to link these two classic cancer markers into one combination diagnostic approach. The Y-box is present in the promoters of genes overexpressed in various cancers, such as breast cancer, colon cancer, and prostate cancer (50–53). Studies have shown that the increase in YBX1 expression or nuclear translocation in cells was accompanied by changes in the mRNA or protein encoded by genes that affect tumors biological function (54, 55). These results indicated that CCAAT is an important oncogene in tumors and that YBX1 is responsible for the activation of the CCAAT oncogene in cancer cells (25, 56). Our data showed that the expression and nuclear translocation of YBX1 in A549 cells were greater than those in H1299 cells. After YBX1 silencing or overexpression, we found synchronous changes in MUC1 at the mRNA and protein levels. However, it cannot be interpreted as YBX1 transcriptionally regulates MUC1 expression because even in the nucleus, YBX1 may affect mRNA levels not only through transcription but also through posttranscriptional regulation to affect mRNA stability (57). We subsequently designed an Act-D transcriptional repression assay, which proved that YBX1 affected MUC1 expression through transcriptional regulation. Based on two potential binding sites, we designed two primers for a ChIP assay and three luciferase plasmids, which proved that YBX1 bound to the -1480-1476 position in the MUC1 promoter region and positively regulated MUC1 transcription.

Our retrospective cohort study of 176 lung adenocarcinoma patients found that low coexpression of YBX1 and MUC1 was the only protective factor for DFS. Therefore, we speculated that YBX1 affects the recurrence and metastasis of lung adenocarcinoma by regulating MUC1. We selected the tumor biological functions related to recurrence and metastasis for verification, including invasion and migration ability and stem-like properties (58–61). Experiments on biological phenotype and related markers, including E-cadherin, β-catenin, Snail (19, 62), and the MMP family (63, 64), showed that MUC1 partly rescued the decreased invasion and migration abilities of A549 cells caused by YBX1 silencing. Therefore, YBX1 and MUC1 are important genes that affect the occurrence, metastasis, and stemness of lung adenocarcinoma *in vitro*.

The mouse xenograft model proved that YBX1 and MUC1 affected tumor volume and weight *in vivo*. Although the IHC staining was weak, there were obvious differences under high magnification, which may be related to the strict unification of DAB color development time to prevent overstraining. YBX1, as a transcription factor, is theoretically highly expressed in the nucleus, and high expression was found in the cytoplasm in our actual IHC staining. We consulted the YBX1 difference in the GeneCards database, showing that extracellular, nucleus, and cytosol all have the highest expression (Confidence:5). The standard pictures on the human protein atlas (http://www.proteinatlas.org), show that cytoplasm YBX1 staining is stronger than nucleus in colon, breast, prostate, and lung cancer, which is similar to our results. The GeneCards also shows the MUC1 expression difference, containing nucleus (5), cytosol (2), and CD133 confidence as below endoplasmic reticulum (5), nucleus (3), cytosol (2). The specific reason for protein location should be related to the partial loss of the subcellular protein antigenic epitope during microwave antigen repair in process of IHC staining.

Then, we injected A549 cells into the tail vein of nude mice to generate a metastasis model, which was a better way to simulate tumor hematogenous metastasis. The changes in YBX1 and MUC1 expression significantly changed the *in vivo* imaging intensity and the number and size of metastases. Two animal models demonstrated that YBX1 and MUC1 were key genes for tumor growth, and blood metastasis *in vivo*.

**Figure 7:**
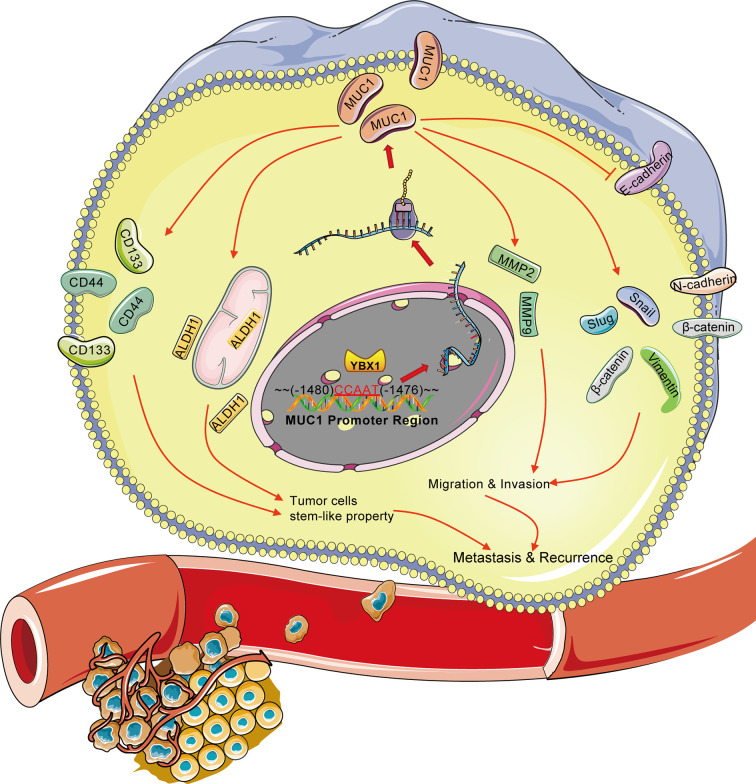
Overview of the transcriptional regulation between YBX1 and MUC1 in lung adenocarcinoma. Activation of MUC1 promoter regions (from -1480 to -1476) by YBX1 promotes the stemness and metastasis of lung adenocarcinoma. The arrow (→) indicates direct or indirect positive regulation. The symbol (┤) indicates negative regulation.

The relationship between these two classic markers is summarized in **Figure 7**. In lung adenocarcinoma cell lines, YBX1 directly or indirectly activates the MUC1 promoter region from-1480 to -1476, this transcriptional regulation targets downstream metastasis and stem-like properties. The effect on metastasis and stemness was reflected not only in the expression of multiple protein markers *in vitro* experiments but also in the effects on tumor growth and blood metastasis *in vivo* (**Figure 7**). Our study verified the high correlation between YBX1 and MUC1 expression in lung cancer for the first time, and the results were consistent with those in large databases. The large correlation between the two classic tumor biomarkers and the strong relationship with the prognosis and recurrence of lung adenocarcinoma patients provided us with a combined diagnosis approach that is different from traditional single tumor biomarkers. The in-depth mechanism of the interrelatedness of the two molecules also provided a possible target for guiding actual clinical treatment to improve patient survival and reduce recurrence.

## Data Availability Statement

The raw data supporting the conclusions of this article will be made available by the authors, without undue reservation.

## Ethics Statement

The studies involving human participants were reviewed and approved by the Medical Ethical Committees of the First Affiliated Hospital of Dalian Medical University. The patients/participants provided their written informed consent to participate in this study. The animal study was reviewed and approved by the Animal Experimental Ethical Committee of Dalian Medical University.

## Author Contributions

All authors contributed to the design of the study and the preparation and critical revision of the manuscript and agreed to be accountable for all aspects of the study. The manuscript is approved by all authors for publication.

## Funding

This study was supported by grants from the National Natural Science Foundation of China (Nos. 81173453, 81774078 and 81803886) and the Natural Science Foundation of Liaoning Province of China (Nos. 201602227 and 20170540300).

## Conflict of Interest

The authors declare that the research was conducted in the absence of any commercial or financial relationships that could be construed as a potential conflict of interest.

## Publisher’s Note

All claims expressed in this article are solely those of the authors and do not necessarily represent those of their affiliated organizations, or those of the publisher, the editors and the reviewers. Any product that may be evaluated in this article, or claim that may be made by its manufacturer, is not guaranteed or endorsed by the publisher.
